# Social and economic costs and health-related quality of life in non-institutionalised patients with cystic fibrosis in the United Kingdom

**DOI:** 10.1186/s12913-015-1061-3

**Published:** 2015-09-28

**Authors:** Aris Angelis, Panos Kanavos, Julio López-Bastida, Renata Linertová, Elena Nicod, Pedro Serrano-Aguilar, BURQOL-RD Research Network

**Affiliations:** Department of Social Policy and LSE Health, London School of Economics and Political Science, Houghton Street, London, WC2A 2AE England; University of Castilla-La Mancha, Talavera de la Reina, Toledo, Spain; Red de Investigación en Servicios Sanitarios en Enfermedades Crónicas (REDISSEC), Madrid, Spain; Canary Islands Foundation for Health and Research (FUNCIS), Las Palmas de Gran Canaria, Spain; Evaluation and Planning Service at Canary Islands Health Service, Santa Cruz de Tenerife, Spain

**Keywords:** Cystic fibrosis, Cost-of-illness, Social cost, Health-related quality of life, UK

## Abstract

**Background:**

This study aimed to determine the societal economic burden and health-related quality of life (HRQOL) of cystic fibrosis (CF) patients in the UK.

**Methods:**

A bottom-up cost-of-illness, cross-sectional, retrospective analysis of 74 patients was conducted aiming to estimate the economic impact of CF. Data on demographic characteristics, health resource utilisation, informal care, productivity losses and HRQOL were collected from questionnaires completed by patients or their caregivers. HRQOL was measured with the EuroQol 5-domain (EQ-5D) instrument.

**Results:**

Using unit costs for 2012 we found that the average annual cost for a CF patient was €48,603, with direct health care costs amounting to €20,854 (42.9 % of total costs), direct non-health care costs being €21,528 (44.3 %) and indirect costs attributable to productivity losses being €6,222 (12.8 %). On average, the largest expenditures by far were accounted for by informal care (44.1 %), followed by medications (14.5 %), acute hospitalisations (13.9 %), early retirement (9.1 %) and outpatient and primary health care visits (7.9 %). Sharp differences existed depending on whether CF patients were in need of caregiver help (€76,271 versus €26,335). In adult CF patients, mean EQ-5D index scores were 0.64 (0.93 in the general population) and mean EQ-5D visual analogue scale scores were 62.23 (86.84 in the general population); among caregivers, these scores were 0.836 and 80.85, respectively.

**Discussion:**

Our analysis highlights the importance of the economic and quality of life consequences of CF from a societal perspective. The results highlight that beyond conventional costs such as acute hospitalisations, medication and outpatient and primary care visits, indirect costs related to informal care and early retirement, have significant societal implications. Similarly, our analysis showed that the average EQ-5D index score of adult CF patients was significantly lower than in the general population, an indication that a methodological bias may exist in using the latter in economic analyses.

**Conclusion:**

CF poses a significant cost burden on UK society, with non-health care and indirect costs representing 57 % of total average costs, and HRQOL being considerably lower than in the general population.

## Background

Cystic fibrosis (CF) is one of the most prevalent, fatal, inherited rare disorders among people of Caucasian descent. The European prevalence ranges between 1/8,000 and 1/10,000 individuals [1]. Data from the UK CF neonatal screening NHS newborn blood spot-screening programme, suggest that 1 in 2,500 babies are born with the disease [2]. The natural course of the disease involves a gradual and progressive deterioration in lung function due to the deranged chloride transport, leading to thick and viscous secretions not only in the lung but also in pancreas, liver, intestine and reproductive tract [3]. Complications due to chronic colonisation by bacteria, lead to destruction of lung architecture and respiratory failure and are the most common causes of death among CF patients [4].

A dramatic improvement in survival among CF patients has been observed in recent years [5] and, as a result, CF is no longer a major mortality cause among children [6, 7]. Despite its low disease prevalence, CF exerts a potentially important economic impact on health care resources, and other social costs [8, 9] and has significant impact on patients’ health-related quality of life (HRQOL) [10–12].

The issue of total costs related to the treatment of CF and the HRQOL for CF patients is poorly understood. Although a number of recent studies have measured independently the economic and HRQOL impact on CF patients [8–16], a comprehensive study in the UK that examines all cost dimensions (direct medical, direct non-medical and indirect costs) and links cost with HRQOL is missing [17].

In this study we report and analyse CF patient-level primary data from the UK collected under the auspices of the BURQOL-RD initiative [18]. The study objectives are twofold: first, to estimate the societal costs of CF by accounting for all direct health, direct non-health care and indirect costs, and, second, to assess the HRQOL of patients with CF.

## Methods

### Research design and sample

This was a bottom-up, retrospective, cross-sectional study of non-institutionalised patients diagnosed with CF, receiving outpatient care. Because of the lack of a publicly available, NHS-based CF registry in the UK, a convenience sample of patients was recruited from the Cystic Fibrosis Trust (CFT) that holds its own anonymised register of patients. The survey was anonymous and patients were contacted by the Trust. A CF diagnosis, non-institutionalised status and membership of CFT determined patient eligibility. Questionnaire responses received by the research team had no identification information (name, address/postcode, e-mail or telephone). All patients and caregivers were informed about the study objective, data confidentiality and were asked to indicate their understanding of the study conditions and agreement to participate. The study protocol was submitted to the London School of Economics (LSE) Research Ethics Committee and received an exemption.

### Information and variables of interest

Following the identification of the patient sample, CFT sent questionnaires electronically and by post to eligible patients at the end of February 2013. The questionnaire comprised two parts, the first identifying costs and the second including HRQOL. The data collection was carried out between end-February and end-May 2013, with reminders sent at the end of April and May. Demographic, clinical and resource use data were collected from CF patients and their caregivers. The questionnaire was detailed enough to reduce either exaggeration or underestimation.

Following receipt of completed questionnaires, patients were divided into two groups: first, high severity or disability, needing caregiver assistance in order to perform basic (dressing, hygiene, eating, etc.) or instrumental (meal preparation, shopping, laundry) daily activities; and, second, low severity or disability, if they did not need such assistance.

### Costing methodology

We used the prevalence approach to estimate resource use and, subsequently, costs from a societal perspective. Disease prevalence takes into account all direct health care resources used for prevention, treatment and rehabilitation, other non-health care resources used (formal and informal care), and indirect costs (productivity loss) within a given year (in a population or in a sample of patients) as a consequence of the illness considered. Prevalence-based cost-of-illness analysis has the advantage of incorporating measurements of total annual health care expenditure, which is particularly relevant for chronic conditions such as CF requiring long-term treatment. In this context, a bottom-up costing approach was used to estimate total and average annual costs.

Data on resource utilisation were collected for each patient and, where appropriate, caregiver. To estimate resource utilisation, the questionnaire solicited information covering the 6-month period prior to the study (12 months for hospital admissions). Data for the preceding 6 months were extrapolated to the entire year. We considered 6 months to be an appropriate recall period [19]. Patients and caregivers were asked about reductions in working time (temporary and permanent sick leave or early retirement), and these data were used to calculate productivity losses. Non-professional caregivers were also asked about informal care time. A list of basic domestic activities (e.g. dressing, bathing, feeding, etc.) and other non-domestic activities (e.g. travelling, shopping, socialising, etc.) was provided, and carers had to specify the approximate daily or weekly time they spent on these activities.

Direct medical costs were derived from health care utilisation. The cost of resources used by patients was calculated based on the relevant unit costs and the average utilisation per patient in the sample. Information about the number of hospital admissions, the number of emergency visits and data for the volume of outpatient care (rehabilitation, medical tests and examinations, visits to health professionals and home medical care) were obtained from the questionnaires.

Unit costs were obtained mostly from the UK payment by results database [20]; additional publicly available resources were used to fill in any remaining data gaps [21, 22]. Unit costs were then multiplied by the respective resource quantities to derive the annual cost per patient, using 2012 as the reference year. In a similar way, resource utilisation information relating to consumption of prescription drugs and medical support devices was obtained from the questionnaires. When no information concerning the number of units per pack was available, we assumed the largest dispensing pack for prescription drugs. Prescription drug unit costs were obtained from the National Drug Tariff database [23] and the British National Formulary [24], whereas unit costs for medical support devices were obtained from major electronic commerce websites.

Direct non-health care costs were the result of aggregating three items: non-health care transportation, social care services (formal care) and caregiver’s time (informal care, provided by non-professional caregivers, who are often relatives, but could also be friends or neighbours and who are not paid for the care provided). Informal care concerned the time spent helping the patient with their basic activities of daily living (ADL), and the time spent helping with necessary instrumental activities of daily living (IADL). The approach used to value care hours was the proxy good method, which values time as an output and values the care provided by the informal caregiver considering that if they did not provide these services, their presence would have to be substituted by a professional caregiver who could provide them [25].

Information on formal (paid) care provided by professional caregivers and other social services was obtained from the questionnaires and is included under the social services category.

Indirect costs were obtained from physical units (days of sick leave and early retirement) converted into monetary units based on the human capital approach [26], using worker gross average earnings from the Annual Survey of Hours and Earnings [27] conducted by the Office for National Statistics to proxy productivity losses.

### Patient and caregiver outcomes

Patient and caregiver outcomes were obtained via the EQ-5D-5 L questionnaire [28], the Barthel Index [29] and the Zarit Burden Interview [30]. The EQ-5D-5 L is a generic instrument of HRQOL, commonly used in economic evaluations and routinely included in health technology assessments. Its five dimensions (mobility, self-care, everyday activities, pain/discomfort and anxiety/depression), enable a total of 245 possible health states to be defined, taking values from 0 (death) to 1 (perfect health). The second part of the EQ-5D consists of a vertical 20-cm, 0–100 Visual Analogue Scale (VAS), where 0 represents the worst and 100 represents the best imaginable health states. Respondents mark a point on the scale to reflect their overall health on the day of the interview [28]. Evaluations of these health states have been reported for the general population [31].

The Barthel Index is widely used to assess physical disability and measures the ability of a person to perform ten basic ADL, obtaining a quantitative estimate of the subject’s degree of dependence. Total possible scores for the UK range between 0 and 20, with lower scores indicating increased disability [29].

Finally, the Zarit Burden Interview (22-item version) measures the subjective burden among caregivers. Each item is a statement to which the caregiver is asked to respond using a 5-point scale, with options ranging from 0 (never) to 4 (nearly always). The total score ranges from 0 to 88, with scores under 21 corresponding to little or no burden and scores over 61 to severe burden [30].

## Results

Of the 234 questionnaires sent, 131 questionnaires (56 %) were returned from CF patients. Of these, 57 questionnaires were excluded because the information they contained was deemed to be insufficient or inadequate. Therefore, the valid sample totalled 74 patient questionnaires.

Table 1 summarises the main characteristics of the sample. Patients were equally divided between adult and non-adult (37 patients each) and average patient age was 18 years; 52.7 % of patients were male and 44.6 % (33 patients) had a caregiver, whose average age was 37.3 years. The total average time spent on informal caregiving, assuming at least one caregiver, was 74.8 hours per week, (3,900 hours per year).

**Table 1:** Sample characteristics of interviewed CF patients (*n* = 74, SD in brackets)

Average age (years)	
All patients	18.3 (15.1)
Adult patients	31.1 (10.1)
Sex	
Male	52.7 %
Female	47.3 %
Is there a caregiver?	
Yes	44.6 %
No	57.4 %
Average age of (principal) caregiver (years)	37.3 (11.6)
Average informal care hours per week (whole sample)	33.4 (52.9)
Average informal care hours per week (if there is a caregiver)	74.8 (56.5)
Health Related Quality of Life (Visual Analog Scale)	62.23 (20.09)
Adult CF patients (*n* = 37)^*a*^	
Visual Analog Scale score for general population^b^	86.84 (14.41)
Main Caregivers for CF patients (*n* = 33)^c^	80.85 (14.68)
Visual Analog Scale score for general population^*d*^	86.56 (13.79)
Health Related Quality of Life (EQ-5D index score)	
Adult CF patients (*n* = 37)^*a*^	0.640 (0.264)
*EQ*-*5D* index score for general population^b^	0.93 (0.15)
Main Caregivers for CF patients (*n* = 33)^c^	0.836 (0.155)
EQ-5D index score for general population^*d*^	0.91 (0.16)

^a^Of the 37 adult patients, 30 filled the HRQoL questionnaire, out of which 25 patients filled the questionnaire themselves, 1 patient filled it using assistance and 4 patients had the questionnaire filled in by someone else

^b^Reflects general population social tariffs/utilities for the respective patients’ age group (i.e. 25–34)

^c^Of the 33 caregivers, 32 filled the HRQoL questionnaire

^d^Reflects general population social tariffs/utilities for the respective caregivers’ age group (i.e. 35–44)

**Fig. 1:**
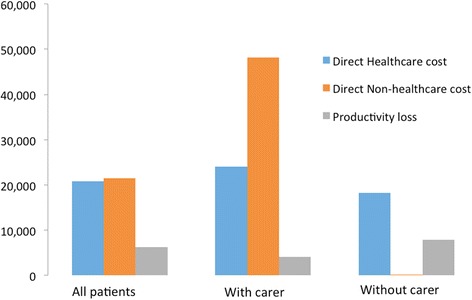
Breakdown of costs according to all CF patients, CF patients with carers, and CF patients without carers (2012, €)

**Fig. 2:**
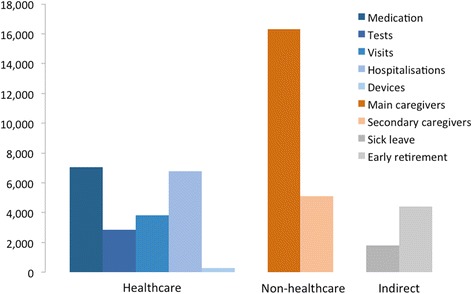
Average costs per CF patient broken down by type of cost (2012, €)

Estimated average annual cost per patient in 2012 was €48,603, and the median was €34,883 (Table 2). Direct non-health care cost was the largest component (44.3 % of the total average cost per patient), followed by direct health care costs (42.9 %) and productivity loss (12.8 %) (Fig. 1). The most important categories of health care costs were medications, (33.8 % of health care and 14.5 % of total costs), followed by acute hospitalizations (32.4 % of health care and 13.9 % of total costs) and outpatient and primary health care visits (18.3 % of health costs and 7.9 % of total costs) (Fig. 2). The most relevant category of direct non-health care cost was informal care, averaging €21,447 (99.6 % of direct non-health care and 44.1 % of total costs), with a special mention given to the cost related to main caregivers (75.8 % of direct non-health care cost and 33.6 % of total costs). Social services only accounted for 0.2 % of direct non-health care cost and 0.1 % of total costs, and non-health care transport represented 0.2 % of direct non-health costs and 0.1 % of total costs respectively. Finally, sick leave accounted for 29 % of productivity loss and 3.7 % of total costs, whereas early retirement accounted for 71 % of productivity loss and 9.1 % of total costs.

**Table 2:** Average annual costs per CF patient (2012, in €)

	Total (*n* = 74)			With carer (*n* = 33)			Without carer (*n* = 41)		
	Mean	±SD	Median	Mean	±SD	Median	Mean	±SD	Median
Direct Health Care Costs									
Prescription medication	7,053.3	(4,737.1)	9,233.3	7,737.3	(4,619.3)	9,795.4	6,502.8	(4,815.3)	8,498.5
Tests	2,865.3	(4,463.7)	1,547.8	2,447.5	(4,124.2)	1,000.3	3,201.5	(4,742.9)	1,866.0
Outpatient & primary health care visits	3,823.3	(4,214.8)	2,513.7	4,224.1	(5,136.4)	2,526.5	3,500.7	(3,328.0)	2,454.2
Acute hospitalisation	6,759.1	(12,532.9)	802.8	9,208.4	(15,590.6)	3,211.4	4,787.7	(9,125.3)	0
Medical devices	287.8	(878.8)	98.7	271.2	(1,007.8)	98.7	301.3	(772.3)	98.7
Health care transportation	64.9	(506.4)	0	131.6	(755.7)	0	11.3	(67.8)	0
Subtotal	*20,853.7*	(*21,336.6)*	15,247.4	*24,020.0*	*(26,057.9)*	15,756.2	*18,305.2*	*(16,513.4)*	14,282.2
Direct Non Health Care Costs									
Non-health care transportation	34.7	(55.6)	15.5	29.6	(32.1)	17.3	38.8	(69.2)	13.8
Social services	46.8	(298.4)	0	35	(200.9)	0	56.3	(360.6)	0
Caregiver time costs (informal care)	21,446.6	(34,034.7)	0	48,092.4	(36,345.7)	38,047.1	0	0	0
Main caregivers	16,323.2	(23,713.8)	0	36,603.5	(22,744.6)	34,831.9	0	0	0
Secondary caregivers	5,123.4	(14,701.3)	0	11,488.8	(20,435.9)	1,286.1	0	0	0
Subtotal	*21,528.1*	*(34,020.5)*	123.4	*48,156.9*	*(36,336.1)*	38,047.1	*95.1*	*(366.5)*	13.8
Total Direct Costs (Direct Health Care Costs & Direct Non Health Care Costs)	*42,381.8*	*(41,307.4)*	*28,254.6*	*72,176.9*	*(43,522.5)*	*65,806.9*	*18,400.4*	*(16,635.9)*	*14,370.3*
Loss of Labour Productivity									
Sick leave	1,805.2	(7,264.1)	0	132.4	(760.4)	0	3,151.7	(9,574.4)	0
Early retirement	4,416.3	(11,248.8)	0	3,961.3	(10,831.6)	0	4,782.6	(11,694.5)	0
Subtotal	*6,221.6*	*(12,772.6)*	*0*	*4,093.7*	*(10,808.3)*	*0*	*7,934.3*	*(14,054.6)*	*0*
TOTAL COSTS	48,603.4	(43,789.6)	34,883.3	76,270.6	(46,073.4)	70,640.8	26,334.6	(25,719.2)	16,591.4

The results differ significantly depending on whether CF patients needed the help of a caregiver. A CF patient with caregiver help had a total average annual cost of €76,271 (median €70,641), compared with €26,335 (median €16,591) for a patient who did not require personal care.

Where a caregiver was present, implying higher severity patients, the most relevant cost category was informal care with an average cost of €48,092 (63.1 % of total costs). Direct medical and direct non-medical costs represented 31.5 and 63.1 % of total costs respectively. In terms of direct medical cost categories, hospitalisations ranked highest (26.2 % of direct medical costs, and 18.2 % of total costs), followed by medications (35.5 % of direct medical costs and 24.7 % of total costs), and outpatient and primary health care visits (19.1 % of direct medical costs and 13.3 % of total costs). Social services only accounted for 0.2 % of direct non-medical costs and 0.1 % of total costs, whereas non-healthcare transport only represented 0.2 % of the direct non-medical costs and a 0.7 % of total costs. Productivity loss accounted for 5.4 % of total costs, with sick leave and early retirement separately accounting for 29 and 71 % of indirect costs, and 3.7 and 9.1 % of total costs respectively.

For patients without a caregiver, direct medical and direct non-medical costs represented 69.5 and 0.4 % of total costs respectively, while indirect costs represented 30.1 % of total costs. The most important categories of direct medical costs were medications (35.3 % of medical costs and 24.7 % of total costs), acute hospitalizations (26.2 % of medical costs and 18.2 % of total costs) and outpatient visits (19.1 % of medical costs and 13.3 % of total costs). Social health services and non-health care transport accounted for 0.2 % and 0.15 % of total costs, respectively. Sick leave and early retirement accounted for 39.1 and 60.3 % of indirect costs and 12 and 18.2 % of total costs, respectively.

Further analysis was conducted to explore the differences in costs between adults and children (Table 3). Average total annual costs per adult and adolescent patient were €44,583 (median €31,511) and €52,624 (€46,356) respectively, of which direct and indirect costs comprised 72.1 (€32,140) and 27.9 % (€12,443) in the adult group, with no indirect costs in the adolescent group. Direct health care costs were much higher than direct non- health care costs among adult patients (€26,439 vs. €5,701), compared with the adolescent patients group, where the opposite trend was observed (€15,268 vs. €37,355), indicating a much higher impact of informal care on total costs in the adolescent group.

**Table 3:** Average annual costs (main groups only) for adult and adolescent CF patients, (2012, in €)

	All patients (*n* = 74)			Adult patients (*n* = 37)			Adolescent patients (*n* = 37)		
	Mean	±SD	Median	Mean	±SD	Median	Mean	±SD	Median
Direct Health Care Costs	20,853.7	(21,336.6)	15,247.4	26,439.3	(21,604.7)	21,944.0	15,268.2	(19,805.3)	9,599.8
Direct Non-Health Care Costs	21,528.1	(34,020.5)	123.4	5,700.8	(13,521.7)	31.1	37,355.4	(40,610.1)	24,313.1
Total Direct Costs	42,381.8	(41,307.4)	28,254.6	32,140.1	(29,596.5)	23,089.7	52,623.5	(48,666.5)	46,355.6
Indirect Costs	6,221.6	(12,772.6)	0	12,443.2	(15,850.7)	1,747.3	0	0	0
Total Costs	48,603.4	(43,789.6)	34,883.3	44,583.2	(38,557.5)	31,511.3	52,623.5	(48,666.5)	46,355.6

With regards to HRQOL of adult patients the EQ-5D index score was 0.64 out of 1, and the EQ-5D visual analogue scale score was 62.23 out of 100 (Table 1). These scores are lower than the EQ-5D values for the UK adult general population (0.93 and 86.84, respectively) after controlling for age [32]. For caregivers, the mean EQ-5D index and VAS scores were 0.836 and 80.85 respectively (Table 1), which are lower than in the UK general population (0.91 and 86.56 respectively). Among adult patients, the average Barthel index was 19.27 reflecting very low dependence, while the average Zarit burden interview score burden was 29.03, indicating a moderate burden for caregivers (Table 1).

## Discussion

In this study we have provided a descriptive rather than quantitative analysis of total cost and HRQOL among patients with CF. Among rare diseases, CF represents a health problem with important societal impact in high-income countries, including the UK [8, 9, 33]. The incidence and prevalence of CF and its health and social impact in terms of mortality, morbidity, economic cost and quality of life justify the attention received from health authorities and society. A recently published systematic review studying the socioeconomic impact of ten rare diseases identified in total 29 costing studies related to CF, four of which investigated aspects of CF management in the UK mainly relating to direct health care costs [17]. The first ever CF costing study in the UK investigated the direct medical costs of patients served at a regional adult CF centre [34]. To date however, this is the first UK-based study attempting to quantify the total (direct medical, direct non-medical and indirect) cost for CF patients together with estimates of quality of life. Studies conducted in different settings prove testament to the high costs associated with CF. For example, annual cost per patient amounted to €41,468 in Germany [33], whereas annual mean direct health care costs per patient were shown to vary across countries. In Germany these were found to range from €17,219 per patient per year, increasing to €21,782 allowing for IV therapy [35]. In the USA, one study showed average annual treatment costs per patient to be $63,127 in 2006 (€50,299) [36], whereas another study suggested a mean annual cost of $43,000 per CF patient in 2008 (€29,378) [15]; however the latter is most likely to be an underestimate mainly because direct non-medical costs were not extensively investigated to the same extent as in our study. In France the direct costs were found to be €22,725 in 2003 [37], whereas in Australia national registry data have indicated that the presence of chronic infections increases cost of care by 70–164 % [16].

The present analysis highlights the importance of studying the economic consequences of CF from a societal perspective and interpreting the results in an international context. Our results provide insights into the distribution of CF costs and their impact on national health expenditure as well as patient and family income. Beyond the average annual total cost of €48,603 (ranging from €26,335 to €76,271 for patients without carers and with carers, respectively), we found that informal care, medication, acute hospitalisations, early retirement and outpatient and primary health care visits represented the highest expenditures.

The high contribution of informal care to the costs identified in this study may have several explanations. First, our methodology may have influenced our estimates. In earlier studies, indirect costs included both job loss and informal care costs. Recently published cost-of-illness studies, however, use more precise classifications of the items that contribute to societal costs. Second, our study design excluded institutionalised patients from the analysis and, therefore, the cost estimates produced in this study are likely to be an under-estimate of the total CF cost due to non-inclusion of institutionalisation and long-term care costs.

HRQOL can be a useful indicator together with other information sources such as incidence, prevalence, mortality and costs to set priorities in health and measure the effectiveness of health interventions on disease management. Our analysis showed that the average EQ-5D index score of adult CF patients was lower than in the general population. Despite its relatively low prevalence, CF is characterized by a substantial economic burden and patients with higher dependence on caregivers, therefore reflecting a higher disability, are more likely to incur higher productivity losses compared to people with lower dependence. Although it would have been of great interest to expand upon the notion of higher disease burden being associated with higher cost by exploring the association of decrements in EQ-5D scores with increases in cost, this would not yield robust estimates because of the relatively small number of patients with available EQ-5D scores in the sample, giving rise to relative few degrees of freedom and, therefore, decreasing the credibility of the results. Instead, the existence or absence of a caregiver was used as a proxy for disability, resulting in a larger sample.

Our study is not without limitations. The first limitation relates to sampling issues. Both the study sample and the recruitment process may limit the external validity of the study. However, other CF studies have used smaller sample sizes due to the low disease prevalence and high rates of participation refusal [35–38]. Although the sample was almost evenly distributed between high- and low-severity patients, we cannot guarantee the avoidance of selection bias but this is common in most rare disease studies involving small numbers of patients. There may also be potential recall bias, given that patient-based data were obtained by questionnaire. A second limitation relates to the non-use of disease-specific HRQOL instruments, such as the cystic fibrosis questionnaire (CFQ). However, a recent systematic review of HRQOL instruments used for rare diseases concluded that the EQ-5D-5 L can be considered a cross-sectional valid generic health outcome measure reflecting the progression of CF [39]. In addition, we have used the Barthel Index and the Zarit scale as proxies to measure disability and severity. Finally, our study used cross-sectional data. An ideal study would be a prospective longitudinal study of a CF cohort, but this type of study was beyond our means and no such study has been undertaken in CF.

Despite the limitations of cost-of-illness analysis studies, governments continue to encourage such research, as the information emerging about the financial impact of disease provides a useful input for program planning and public policy design. This information complements epidemiological data on population-level health problems. This study represents the most complete and realistic costing to date of the burden of CF performed in the UK, a key strength being the use of a bottom-up approach to costing. Additionally, estimating costs over a one-year period has provided a more accurate picture of the medium-term burden of CF.

## Conclusion

By pursuing a bottom-up cost and HRQOL study, we have shown that direct health care costs of CF are substantial, although other social costs, such as informal care, are even higher proportionately, and that higher disability, as reflected through the existence or absence of a caregiver, is associated with significantly higher CF social costs. Overall, CF represents a significant hidden cost to society and this should be taken into account when considering treatments and support programs for CF patients and their caregivers. The data in this study could form the basis for integrated and harmonised approaches to periodically assess the future impact of new public policies and interventions for rare diseases at national and EU level.

## Appendix I

Burqol-Rd Research Network

- Canary Islands Foundation for Research and Health (FUNCIS) (Spain): Pedro Serrano-Aguilar, Renata Linertová
- Universidad Castilla-La Mancha (Spain): Julio López-Bastida, Juan Oliva-Moreno
- Research Institute for Rare Diseases, Instituto de Salud Carlos III (Spain): Manuel Posada de la Paz, Manuel Hens Pérez, Ignacio Abaitua
- National Center for Rare Diseases, Istituto Superiore di Sanità (Italy): Domenica Taruscio, Yllka Kodra
- Mario Negri Institute for Pharmacological Research (Italy): Arrigo Schieppati
- Bulgarian Association for Promotion of Education and Science (Bulgaria): Rumen Stefanov, Georgi Iskrov
- Centre for Public Affairs Studies Foundation (Hungary): László Gulácsi, Márta Péntek
- Federación Española de Enfermedades Raras (Spain): Rosa Sánchez de Vega García, Claudia Delgado
- London School of Economics and Political Science (UK): Panos Kanavos, Aris Angelis, Elena Nicod
- Leibniz University Hannover (Germany): Johann-Matthias Graf von der Schulenburg, Alexander Kuhlmann
- The Swedish Institute for Health Economics (Sweden): Ulf Persson, Ola Ghatnekar
- University Paris Est (France): Karine Chevreul, Karen Brigham
- Universita Commerciale “Luigi Bocconi” (Italy): Giovanni Fattore, Marianna Cavazza
